# Education Research: Neurologic Education in Physician Assistant Programs

**DOI:** 10.1212/NE9.0000000000200029

**Published:** 2022-12-01

**Authors:** Daniel S. Harrison, Margaret Naclerio, Kathryn Swider, Carl Garrubba, Andrew T. Yu, Andrew Busler, Lena Liu, Christopher Doughty

**Affiliations:** From the Harvard Medical School (D.S.H., L.L., C.D.); Department of Neurology (D.S.H., M.N., A.B., L.L., C.D.), Brigham and Women's Hospital; Department of Neurology (D.S.H., K.S., L.L.), Massachusetts General Hospital, Boston; Department of Physician Assistant Studies (C.G.), Dominican University of California, San Rafael; and Department of Neurology (A.T.Y.), University of California San Francisco Medical Center.

## Abstract

**Background and Objectives:**

A growing number of advanced practice providers (APPs) are entering neurologic practice, and educational initiatives focused on postgraduate training in neurology for these providers are growing in turn. Neurologic education in APP degree programs is not well defined, which limits the ability to tailor these initiatives to the specific needs of APPs. We aim to describe neurologic education in physician assistant (PA) degree programs to better inform these efforts.

**Methods:**

The 2018 American Academy of Neurology clerkship director survey was adapted for directors of PA programs via an iterative approach. The survey was distributed to program directors (PDs) of accredited programs in Fall 2021 and again in Spring 2022 for nonresponders. Simultaneously, websites of accredited programs were systematically reviewed for content related to neurologic education.

**Results:**

Sixty of 255 contacted PDs completed the survey (23.5%). All PDs reported education in selected neuroscience topics. Neuroradiology instruction was included less frequently (66.7%) than neuroanatomy (91.7%) or neurologic examination techniques (95.0%). Twenty-six PDs (43.3%) reported a dedicated neuroscience course; 53 of 260 websites reviewed identified dedicated neuroscience courses (20.8%, *k* = 0.41). Directors of 10 (38.5%) reported neuroscience courses were neuroscience trained. Only 1 program required a neurology clinical rotation in both the website review (0.4%) and the PD survey (1.7%, *k* = 1.00). Elective neurology rotations were offered by 51 programs (85.0%) and used by less than 20% of students in 46 programs (92.0%). More programs with dedicated neuroscience didactics (80.0% vs 74.2%) and offerings in clinical neurology (78.7% vs 66.7%) reported graduates pursuing careers in neurology, but these differences were not statistically significant.

**Discussion:**

Survey respondents reported the inclusion of most of the queried preclinical neuroscience topics, typically distributed throughout the curriculum. Dedicated neuroscience courses were less common and most commonly not taught by a neurologist or neurology APP. Clinical neurology rotations are almost never required, but most programs offer an elective. These results suggest opportunities for augmenting neurologic education in APP degree programs, including encouraging students to take clinical neurology rotations and increasing exposure to APPs practicing neurology. These findings additionally inform key targets for postgraduate educational initiatives.

There are now over 1,000 physician assistants (PAs) working in neurology in the United States, representing a 33.4% increase compared with 2016.^1^ As the number of PAs choosing careers in neurology continues to grow, so too does the importance of ensuring these providers receive educational support to succeed in practice. Advanced practice providers (APPs) report limited neurologic education in their undergraduate degree programs, specifically identifying neuroradiology, neuroanatomy, and neurologic differential diagnosis as key knowledge gaps.^2,3^ Efforts are already underway to enhance on-the-job training for recently graduated APPs, and some institutions have even created residency and fellowship programs for APPs interested in careers in neurology.^2,4,5^ The success of these programs and the utility of educational resources developed for APPs starting careers in neurology critically depends on an accurate understanding of the educational needs of this population. Although APPs may fill similar roles in neurology practice, there are key differences in PA and NP training programs. Notably, there are multiple tracks in NP programs, which makes these programs somewhat more heterogenous when compared with PA programs. The Physician Assistant Education Association publishes a curriculum report based on survey data from program directors (PDs), but it does not contain information regarding neurologic education.^6,7^ The National Commission on Certification of Physician Assistants (NCCPA) provides a blueprint for the PA National Certifying Exam (PANCE) broken down by medical specialty, but this contains only a list of tested neurologic diseases.^8^ We were unable to identify a more systematic review of neurologic education in PA programs. In the present study, we therefore aim to describe neurologic education in accredited PA programs, identify strengths and opportunities for improvement in neurologic education, and determine the feasibility of website review for collecting information regarding neurologic education in APP programs to supplement survey data.

## Methods

The American Academy of Neurology previously distributed a survey to neurology medical school clerkship directors to better define neurologic education of US medical students.^9^ We adapted this survey for PDs of PA programs through an iterative approach, incorporating input from APP and MD educators and providers. The survey instrument is available in the supplement (eAppendix 1, links.lww.com/NE9/A8). We included questions about preclinical and clinical neurology experiences and instruction. To understand how neurology was prioritized in comparison to other nonsurgical specialties, we chose to directly compare preclinical education in neurology to preclinical education in cardiology. We specifically chose cardiology because, like neurology, its practice requires proficiency in anatomy, physiology, examination techniques, and imaging and because it is important for general practitioners. PDs of accredited or provisionally accredited PA programs with students enrolled as of Fall 2021 with publicly available email addresses were invited to participate. Nonresponders were contacted again in Spring 2022.

Websites of PA programs within the United States currently holding continued or provisional accreditation status from the Accreditation Review Commission on Education for the Physician Assistant with enrolled students as of Fall 2021 were systematically reviewed following a detailed rubric, which is available in the supplement (eAppendix 2, links.lww.com/NE9/A8). Course lists and descriptions from program websites were reviewed for presence of dedicated neuroscience didactic courses, course content, and presence of available clinical neurologic exposure. Duration of neuroscience didactics was compared with course hours of cardiovascular didactics. Data were recorded in an Excel spreadsheet and reviewed by D.S.H. and M.N. for consistency.

Quantitative results are reported using simple, descriptive statistics, except as noted below. Agreement of results from website review and PD survey data are reported using Cohen κ statistic. Free-text, open-ended survey responses regarding neurologic examination education were analyzed qualitatively in Excel by D.S.H. using an inductive approach.

### Standard Protocol Approvals, Registrations, and Patient Consents

This study was deemed to be of minimal risk and thus received an exemption from full review from the Mass General Brigham Institutional Review Board.

### Data Availability

Anonymized data not published within this article will be made available by request from any qualified investigator.

## Results

A total of 260 accredited PA programs with enrolled students were identified as of Fall 2021, all of which were included in the website review. Of these programs, contact information was publicly available for 256 programs directors or administrators. One PD was not invited to participate because he contributed to survey design. Overall, 60 PDs completed the survey (response rate 23.5%).

### Preclinical Neuroscience Education

Five program websites did not have sufficient information to determine whether a dedicated neuroscience course was included in the curriculum. Of the remaining 255 program websites, 53 (20.8%) indicated presence of a dedicated neuroscience course. By contrast, 26 of 60 PDs (43.3%) reported a dedicated neuroscience course in their program on the survey. There were 56 programs with both a website that included information about courses included in their preclinical curricula and a PD who completed the survey. Comparing these sources of data, 14 of 56 websites (25.0%) and 23 of 56 PD survey responses (41.1%) indicated presence of a dedicated neuroscience course (*k* = 0.41).

**Figure 1:**
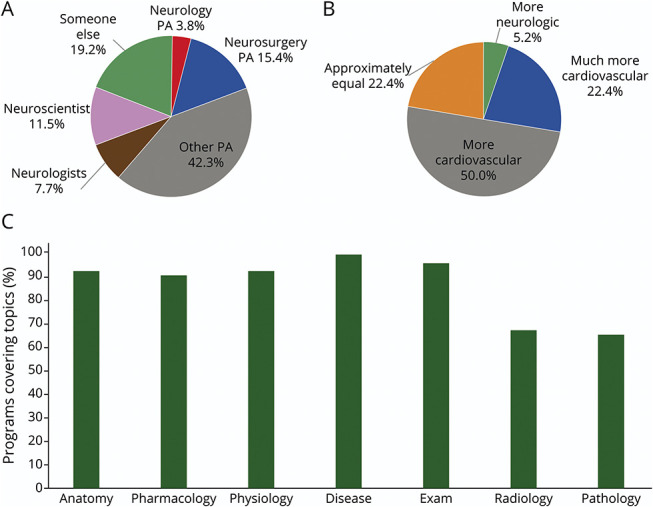
Characteristics of Didactic Neuroscience Education (A) Training of course director for dedicated neuroscience didactic. (B) Program director (PD) comparison of course hours dedicated to didactic cardiovascular vs neurologic education. (C) Percentage of physician assistant (PA) programs including selected topics in neuroscience in the didactic phase.

When a dedicated neuroscience course was present, PDs reported that it was not taught by an individual with a masters or doctoral level degree specialized in neuroscience, neurology, or neurosurgery in 16 programs (61.5%, Figure 1A). All PDs reported offering instruction in selected topics in neuroscience, regardless of presence of a dedicated neuroscience course. PDs reported course content nearly always included neuroanatomy (n = 55, 91.7%), neuropharmacology (n = 54, 90.0%), neurophysiology (n = 55, 91.7%), neurologic disease (n = 59, 98.3%), and neurologic examination (n = 57, 95.0%). Instruction in neuroradiology (n = 40, 66.7%) and neuropathology (n = 39, 65.0%) was often included (Figure 1C). Compared with neurologic education, 42 PDs (72.4%) reported dedicating more course hours to cardiovascular education. Three PDs (5.2%) reported dedicating relatively more time to neurologic education (Figure 1B). Of the program websites that listed course hours for both neuroscience and cardiovascular courses, none listed more course hours dedicated to neuroscience education.

### Neurologic Examination Education

Detailed information on neurologic examination education was not included on program websites. Most PDs reported neurologic examination teaching through a combination of methods. Most often, these were hands-on experience (n = 48, 81.4%) and lecture-based formats (n = 40, 67.8%). Hands-on experience involved students practicing the examination on one another, actors, or patients. Larger group lecture-based formats often included demonstration of examination technique. A smaller majority (n = 30, 50.8%) of respondents indicated that their neurologic examination education also included some form of skills evaluation such as a practical examination, objective structured clinical examination, or skills check-off. A minority of programs (n = 15, 25.4%) indicated that at least some component of neuro examination education was delivered via self-directed resources such as prerecorded videos or textbooks. Very few PDs reported who taught the examination, the level of detail in which the examination was taught, or how much time was dedicated to teaching the examination.

### Clinical Neurologic Education

**Figure 2:**
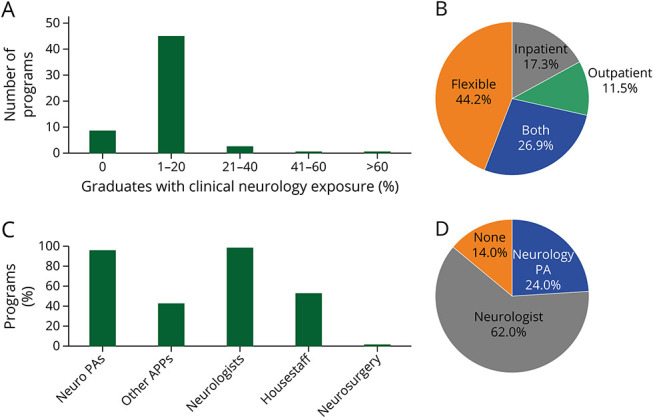
Characteristics of Clinical Neurology Education (A) Percentage of graduates with clinical neurology exposure by program. (B) Setting of the clinical neurology rotation. (C) Providers with whom students work during their neurology rotation by program. (D) Primary preceptor of neurology rotation.

A total of 234 program websites contained sufficient information to determine whether a required neurology clinical rotation was included in the curriculum. Survey data and website review identified 1 program with a required neurology rotation (1.7%, 0.4%, *k* = 1.0). More detailed information about the content of clinical neurologic education was unavailable through website review. Fifty-one PDs (85.0%) reported that elective neurology rotations were offered by their program through the survey. For those programs that offer a neurology elective, 100% of PDs reported that at least 1 student takes the elective each year (Figure 2A). Forty-six of these PDs (92.0%) reported that less than 20% of students take these electives. All PDs indicated that students worked with neurologists on their neurology clinical rotations, and 49 (96.1%) reported that students worked with neurology PAs (Figure 2B). Thirty-seven PDs (71.2%) reported that clinical rotations were either flexible or a mixture of both inpatient and outpatient exposure (Figure 2C). The primary preceptor role in PA programs is roughly analogous to the clerkship director role in medical school. Thirty-one PDs (62.0%) reported that this role was filled by a neurologist. Seven PDs (14.0%) reported that their neurology rotation did not have a primary preceptor (Figure 2D).

### Characteristics of Programs With Graduates Pursuing Careers in Neurology

**Figure 3:**
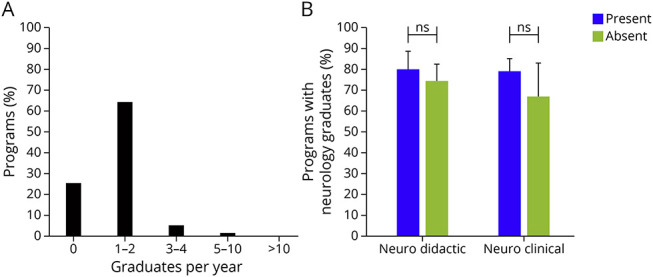
Characteristics of Programs With Graduates Pursuing Careers in Neurology (A) Percentage of programs with graduates pursuing neurology. (B) Exploratory analysis of factors associated with programs reporting graduates in neurology. ns = not significant

PDs reported an average of 40.5 graduates per year. Forty-two programs (73.7%) reported that at least 1 graduate pursues a career in neurology in a typical year (Figure 3A). An exploratory analysis was performed to identify factors associated with programs having graduates pursuing careers in neurology. Twenty programs (80.0%) with compared with 23 programs (74.2%) without dedicated neuroscience didactics reported having graduates in neurology (χ^2^ = 0.26, *p* = 0.61; Figure 3B). Thirty-seven programs (78.7%) with compared with 6 programs (66.7%) without clinical neurology offerings reported having graduates in neurology (χ^2^ = 0.62, *p* = 0.43).

## Discussion

Understanding exposure to neuroscience and neurology in PA programs may be important in designing educational interventions, onboarding programs, or other training programs for early career neurology PAs to address areas of highest need. Here, we report a review of neurologic education in PA programs.

Survey respondents almost universally reported that their preclinical curricula covered content in neuroanatomy, neuropharmacology, neurophysiology, neurologic disease, and the neurologic examination. Neuroradiology and neuropathology were less consistently included in PA program curricula. Whereas only a small group of neurology PAs might be expected to use neuropathologic knowledge in their everyday practice, many will order and review neuroimaging routinely throughout their careers. Recent data from focus groups has suggested that neuroimaging is a key knowledge gap for APPs practicing neurology.^2^ This study adds to the mounting evidence that neuroradiology should be a target of postgraduate education for PAs. Although new graduates of PA programs should not be expected to be experts in interpreting neuroimaging, addition of neuroimaging basics to PA program curriculum, including indications for obtaining neuroimaging, may also benefit graduates practicing in specialties such as primary care and emergency medicine. Most PDs reported more time dedicated to cardiovascular education than neurologic education, despite the fact that foundational anatomy and physiology concepts, key examination maneuvers, and the use of imaging are all critical for understanding both fields. PDs were not asked to explain this; however, one possibility would be that the PANCE contains almost twice as much cardiovascular as neurologic content.^8^ The NCCPA reports that, as of 2020, there were 3,043 PAs practicing cardiology and only 1,007 PAs practicing neurology.^1^ This may be in part due to the shortage of cardiologists in the United States; however, this shortage is comparable to the shortage of neurologists.^10^

Our findings suggest that neuroscience topics are typically distributed throughout preclinical training, as dedicated neuroscience courses were reported in a minority of programs. Neuroscience courses when present were most often directed by individuals who were not specialists in the neurosciences. Although this suggests that neither that these topics are not well taught nor that these courses are of poor quality, it does suggest a potential lack of mentorship from neurology APPs and neurologists for PA students. Furthermore, only 1 PA program required a clinical neurology rotation, and a small number do not routinely offer neurology electives. PDs were not asked why their program did not offer a neurology elective, but our results argue against a lack of interest from students causing this, as each program that offered a neurology elective reported that at least some students chose to take it. It is therefore possible that students in some programs do not have access to a neurologist or neurology PA. There is a clear opportunity for neurologists and neurology PAs to take a more active role in both didactic and clinical neurology education for PA students. This lack of clinical neurology exposure necessarily precludes students from understanding how basic neuroscience topics (such as neuroanatomy) translate into neurologic practice. This lack of basic science/clinical integration has been identified as a major cause of neurophobia among medical students.^11,12^ Whether this disconnect in PA education may also be fueling neurophobia among PA students warrants further study, as this could serve as an important barrier for PAs who might otherwise consider a career in neurology.

One limitation of our study is the low survey response rate among PDs. The study may have been underpowered to detect factors associated with PA programs with students pursuing careers in neurology due to this low response rate. Data acquired via the survey may also be subject to selection bias as a higher proportion of dedicated neuroscience courses were reported via survey vs website review. Complimentary website review was valuable insofar as it allowed us the opportunity to collect information about a higher number of PA programs. For example, website review corroborated the lack of required clinical neurology rotations reported by PDs in the survey. However, website review did not offer more granular detail about education in PA programs. Specifically, website review did not allow for collection of data regarding topics in preclinical neurologic education, neurologic examination education, course director information, clinical neurology electives, student exposure to neurologists and neurology PAs, primary preceptors, or programs with graduates pursuing careers in neurology. Furthermore, when describing the same programs, there was only moderate agreement between the proportion of PDs reporting and the proportion of program websites listing dedicated neuroscience didactics. One possible explanation for this could be that websites were not up to date or offered incomplete information. Taken together, these data suggest that website review may bolster survey data but should not be pursued in isolation for studies of other health professions programs.

Additional limitations of this study include the selection of the PD as the participant. This person, who oversees all didactic and clinical education for a PA program, may not have the most granular information about neurologic education. However, neurologic course directors and primary preceptors were not present in every program and their contact information was rarely publicly available, limiting ability to survey program staff with potentially more detailed information. Our survey was not anonymous, also introducing the possibility of response bias. Finally, because data were obtained via a survey rather than a semistructured interview, qualitative responses could not be explored in more detail than initially offered by the participant.

PA programs offer didactic neuroscience education covering most selected topics, typically distributed throughout the preclinical curriculum. More didactic time is dedicated to cardiovascular than neurologic education. When dedicated neuroscience courses are present, they are occasionally directed by neurologists or neurology APPs. Clinical neurology is most often offered only on an elective basis. Among programs assessed via a survey, most PA students do not participate in clinical neurology electives, and a small number of programs have no opportunity for clinical neurology rotations. Website review alone is likely not sufficient to accurately assess nuances of neurologic education in medical training programs. Review of neurologic education in APP programs should be pursued on a regular basis to identify opportunities for intervention in APP programs and targets for educational initiatives for APP program graduates.

## Appendix Authors

**Table:**

Name	Location	Contribution
Daniel S. Harrison, MD	Harvard Medical School; Department of Neurology, Brigham and Women's Hospital; Department of Neurology, Massachusetts General Hospital, Boston	Drafting/revision of the manuscript for content, including medical writing for content; major role in the acquisition of data; study concept or design; and analysis or interpretation of data
Margaret Naclerio, MPAS	Department of Neurology, Brigham and Women's Hospital, Boston, MA	Drafting/revision of the manuscript for content, including medical writing for content; major role in the acquisition of data; study concept or design; and analysis or interpretation of data
Kathryn Swider, MSN, AGACNP-BC	Department of Neurology, Massachusetts General Hospital, Boston	Drafting/revision of the manuscript for content, including medical writing for content; major role in the acquisition of data; and study concept or design
Carl Garrubba, MPA, DMSc	Department of Physician Assistant Studies, Dominican University of California, San Rafael	Drafting/revision of the manuscript for content, including medical writing for content, and study concept or design
Andrew T. Yu, MD	Department of Neurology, University of California San Francisco Medical Center	Drafting/revision of the manuscript for content, including medical writing for content, and major role in the acquisition of data
Andrew Busler, MSN, ACNP-BC	Department of Neurology, Brigham and Women's Hospital, Boston, MA	Drafting/revision of the manuscript for content, including medical writing for content, and major role in the acquisition of data
Lena Liu, MD	Harvard Medical School; Department of Neurology, Brigham and Women's Hospital; Department of Neurology, Massachusetts General Hospital, Boston	Drafting/revision of the manuscript for content, including medical writing for content, and major role in the acquisition of data
Christopher Doughty, MD	Harvard Medical School; Department of Neurology, Brigham and Women's Hospital, Boston	Drafting/revision of the manuscript for content, including medical writing for content; study concept or design; and analysis or interpretation of data

## Glossary

APP: advanced practice provider
NCCPA: National Commission on Certification of Physician Assistants
PA: physician assistant
PANCE: PA National Certifying Exam
PD: program director
